# Characterization of a Single-Capture Bright-Field and Off-Axis Digital Holographic Microscope for Biological Applications

**DOI:** 10.3390/s25092675

**Published:** 2025-04-23

**Authors:** Jian Kim, Álvaro Barroso, Steffi Ketelhut, Jürgen Schnekenburger, Björn Kemper, José Ángel Picazo-Bueno

**Affiliations:** 1 Biomedical Technology Center, University of Muenster, Mendelstr. 17, D-48149 Muenster, Germany; jian.kim@uni-jena.de (J.K.); alvaro.barroso@uni-muenster.de (Á.B.); ketelhut@uni-muenster.de (S.K.); schnekenburger@uni-muenster.de (J.S.); bkemper@uni-muenster.de (B.K.); 2 Department of Optics, Optometry and Vision Science, University of Valencia, c/Dr. Moliner 50, 46100 Burjassot, Spain

**Keywords:** digital holographic microscopy, quantitative phase imaging, biomedical imaging, label-free multimodal imaging, digital image processing, interferometric microscopy, bright-field microscopy

## Abstract

We present a single-capture multimodal bright-field (BF) and quantitative phase imaging (QPI) approach that enables the analysis of large, connected, or extended samples, such as confluent cell layers or tissue sections. The proposed imaging concept integrates a fiber-optic Mach–Zehnder interferometer-based off-axis digital holographic microscopy (DHM) with an inverted commercial optical BF microscope. Utilizing 8-bit grayscale dynamic range multiplexing, we simultaneously capture both BF images and digital holograms, which are then demultiplexed numerically via Fourier filtering, phase aberration compensation, and weighted image subtraction procedures. Compared to previous BF-DHM systems, our system avoids synchronization challenges caused by multiple image recording devices, improves acquisition speed, and enhances versatility for fast imaging of large, connected, and rapidly moving samples. Initially, we perform a systematic characterization of the system’s multimodal imaging performance by optimizing numerical as well as coherent and incoherent illumination parameters. Subsequently, the application capabilities are evaluated by multimodal imaging of living cells. The results highlight the potential of single-capture BF-DHM for fast biomedical imaging.

## 1. Introduction

In cell biology, label-free quantitative optical microscopy techniques enable advanced, minimally invasive imaging and analysis of living cells as well as dynamic cellular processes. Methods like bright-field (BF) microscopy [1], Zernike phase contrast [2], differential interference contrast (DIC) [3], and digital holographic microscopy (DHM) [4] provide imaging of native cell populations without the requirement for external markers, which may alter the investigated sample. Off-axis DHM permits the fast retrieval of quantitative information about cell morphology and refractive index [5,6,7,8], utilizing quantitative phase imaging (QPI) by precisely measuring the optical path length delays that are caused by a semitransparent specimen against its surrounding medium [9,10]. Moreover, DHM offers QPI with interferometric precision and enhanced image processing, such as multi-focus imaging and extended depth of field by numerical refocusing capabilities [11,12,13]. Various biomedical applications of DHM and QPI have been demonstrated so far, for example, in cancer research [14,15,16], in imaging and quantification of infectious-related effects [17,18,19], as well as in cytotoxicity testing [20,21,22], among others [23,24,25,26,27,28].

DHM can be integrated into multimodal systems to provide complementary information about cellular processes. For example, DHM has been successfully combined with fluorescence or Raman microscopy [29,30,31,32,33,34,35,36,37,38,39,40]. DHM systems with fluorescence capabilities offer additional molecular specificity. However, staining with fluorescent dyes requires additional sample preparation efforts, can lead to cell alterations, and limits long-term time-lapse imaging of living cells, e.g., by phototoxic effects. The combination of DHM with Raman spectroscopy provides additional chemical information in a label-free manner but is limited in spectral signal-to-noise-ratio and acquisition speed, restricting high throughput applications. In addition, both Raman and fluorescence-based imaging require additional, partially expensive optical components and an exposure of the sample to high light intensities. Multimodal systems combining white-light BF microscopy and laser light-based DHM offer label-free, minimally invasive, high-throughput imaging of biological specimens in transmission, with minimum sample preparation demands at low light intensities. This enables fast, full-field imaging, long-term observation of living cell cultures, as well as the analysis of dynamic cellular processes such as motility and proliferation of native samples. Furthermore, BF-DHM is compatible with commercial optical microscopes, which simplifies the utilization in practice for routine applications [41,42,43]. In the investigation of cell cultures, the combination of BF and DHM offers several advantages. For example, it enables the colocalization of absorbance and phase changes in intracellular organelles. It also helps to locate small absorbing structures and allows a pre-identification of sample regions of interest for QPI analysis. Additionally, QPI image-based analysis facilitates the obtention of biophysical cell features such as refractive index (RI), volume, and dry mass [44].

In earlier studies, combined BF-DHM setups were implemented for sequential BF image and digital hologram acquisition [41,42]. However, sequential capturing of images reduces acquisition speed and requires precise synchronization between the digital image recording sensors and the different light sources used for the illumination of the sample [41]. Simultaneous BF and DHM QPI image acquisition was also achieved by employing multiple digital cameras, which increases the complexity of the experimental setup and necessitates precise alignment of the complementary recorded fields of view (FOVs) [45]. More recently, to address these limitations, single-capture bright-field and spatially multiplexed interferometric microscopy (SC-BF-SMIM) has been developed, providing single-camera-based simultaneous QPI and BF imaging by employing a grayscale dynamic range off-axis multiplexing approach [43]. Although effective, SC-BF-SMIM requires a clear reference region in the investigated sample, preventing its application to extended samples such as densely grown cell populations or tissue sections. Furthermore, SC-BF-SMIM uses a Ronchi ruling for interferometric recording, causing two main issues: (1) reduced emission power efficiency, requiring stronger light sources and increasing phototoxicity risk, and (2) the capture of an extra BF reference image, which reduces both the camera’s dynamic range and BF image contrast.

Here, we present the implementation of a single-capture, Mach–Zehnder interferometer-based BF-DHM approach that prevents the need for sequential implementation and multiple camera acquisitions. The proposed BF-DHM concept also overcomes issues associated with Ronchi rulings [43] and enables the analysis of large, connected, or extended samples, such as confluent cell layers or tissue sections. Furthermore, the proposed multimodal reconstruction method is less demanding in terms of spatial frequency and dynamic range multiplexing compared to other single-camera multiplexing approaches [31,32,46,47], making it particularly suitable for high-resolution analysis of partially absorbing samples. The system is based on a commercial inverted BF microscope with an attached DHM module that includes a fiber-optic, off-axis, double-path Mach–Zehnder interferometer. The proposed method provides optimized QPI and BF image recovery simultaneously by utilizing a refined version of the SC-BF-SMIM algorithm. In addition, we present for the first time a systematic characterization of the multimodal system to achieve optimized imaging performance under various bright-field and laser light illumination conditions. Finally, the performance of the optimized system is demonstrated by multimodal imaging of living adherent cells as well as living cells in flow, illustrating its application potential as a robust and fast tool for minimally invasive quantitative live-cell imaging.

## 2. Materials and Methods

### 2.1. Multimodal Microscopy System

**Figure 1:**
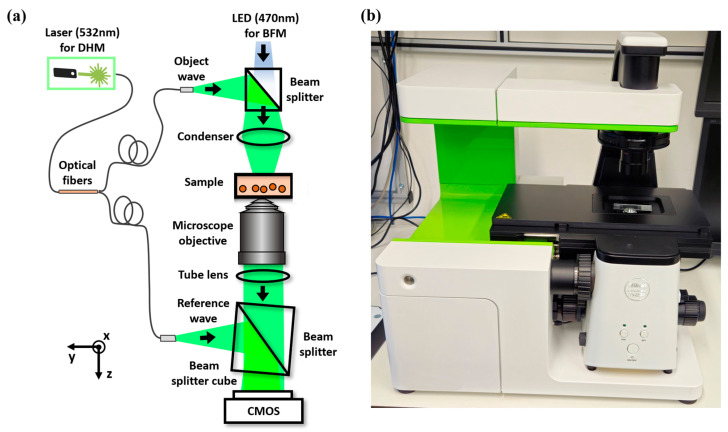
(**a**) Schematic of the multimodal BF-DHM system. LED, light-emitting diode; CMOS, complementary metal–oxide–semiconductor. (**b**) Photo of the BF-DHM system.

Figure 1 presents a sketch of the concept of the multimodal BFM-DHM system, highlighting the components in the setup for simultaneous QPI and BF imaging. The system consists of a commercial inverted BF microscope (ECLIPSE Ts2R, Nikon, Tokyo, Japan), which is additionally equipped with an off-axis DHM module [48] designed to enable dual-mode QPI and BF imaging. For incoherent BF illumination, a light-emitting diode (LED) (M470D4, λ = 470 nm, Δλ = 28 nm, Thorlabs Inc., Newton, MS, USA) is applied. Imaging is conducted using a 20× microscope objective (CFI Plan-Achromat, NA 0.40, Nikon, Japan), together with the tube lens integrated into the microscope. The system is also equipped with a motorized microscope stage (Märzhäuser, Wetzlar, Germany) for precise and automated sample positioning.

The off-axis DHM module is based on a Mach–Zehnder interferometer. Coherent illumination is achieved by a fiber-coupled solid-state laser (Cobolt 06-DPL, λ = 532 nm, Cobolt AB, Solna, Sweden). This laser light is divided by a fiber optic splitter to create the object illumination (O) and reference (R) waves, which are then introduced into the optical path of the microscope via non-polarizing beam splitter cubes. The object wave enters between the LED and the condenser for bright-field illumination, while the reference wave is introduced after the tube lens. An off-axis holographic configuration is obtained by tilting the beam splitter located behind the tube lens to create a slight angle (1.9°) between the object and reference waves, enabling the simultaneous capture of BF intensity images and off-axis holograms at the output port. The system employs a grayscale dynamic range multiplexing approach and a CMOS monochrome sensor (UI-3260CP-M-GL, IDS Imaging Development Systems GmbH, Obersulm, Germany) to record these multiplexed images at 47 fps. The sensor area has a resolution of 1936 × 1216 px and a pixel size of 5.86 µm, resulting in an image size of 11.34 × 7.13 mm^2^ and a FOV of 567 × 357 µm^2^. Please note that the off-axis interferogram fully covers the CMOS sensor. Finally, the recorded multiplexed images are transferred via a USB 3.0 interface to a notebook computer (HP Pavilion Plus Laptop with 13th Gen Intel(R) Core(TIM) i7-13700H processor, Intel, Santa Clara, CA, USA, 2.40 GHz) for automated reconstruction and evaluation with fast Fourier transformation and numerical image subtraction procedures, and utilizing specific software (MATLAB R2024a), for fast (<1 s) recovery of BF and QPI image data.

### 2.2. Multimodal Image Recovery

The grayscale multiplexed images provided by the system consist of a BF image that is superimposed with an off-axis hologram [43]:

(1) $$I\;\propto \;BFI+{\left|O\right|}^{2}+{\left|R\right|}^{2}+RO^{*}+OR^{*},$$

where BFI is the BF image intensity, O and R are the complex amplitudes of the object and reference waves, respectively, ∝ is the proportional symbol, and * denotes the complex conjugate.

**Figure 2:**
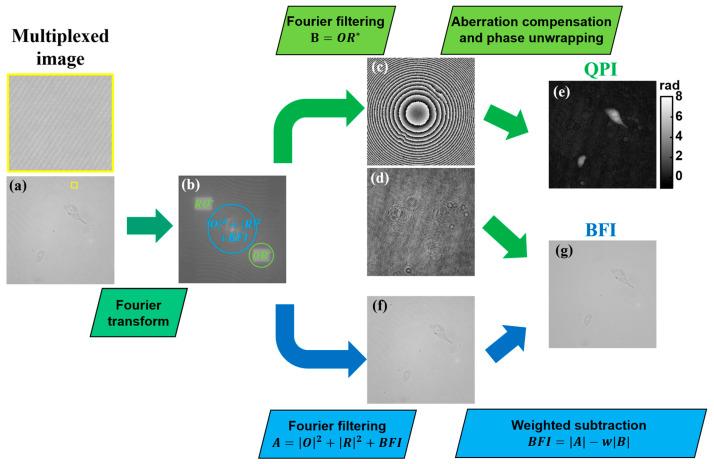
Flowchart of the numerical process for separated obtention of QPI and BF images for the example of adherent pancreatic tumor cells. (**a**) Multiplexed image recorded by the multimodal system. (**b**) Fourier transform of (**a**), highlighting the location of the different holographic diffraction orders and spectral information of the BFI. (**c**,**d**) Phase and intensity images reconstructed by Fourier filtering of the cross-correlation term (OR*) (see green circle in (**b**)). (**e**) Recovered QPI image from (**c**) after phase aberration compensation and phase unwrapping application. (**f**) Intensity image recovered from Fourier filtering of the central spectral region (see blue circle in (**b**)). (**g**) Resulting BF image after weighted subtraction of (**d**,**f**). O and R, complex amplitudes of the object and reference waves, respectively; BFI, bright-field image; QPI, quantitative phase image; w, weight factor. Yellow-marked enlarged image is included in (**a**) to better show the interferometric carrier fringes in the multiplexed image.

Equation (1) shows that different imaging mode components are multiplexed in the recorded light intensity, which requires a demultiplexing recovery process to separate and retrieve the QPI and BF images. Figure 2 illustrates the image recovery process by living adherent pancreatic tumor cells, adapting a modified version of the previously reported SC-BF-SMIM reconstruction method [43]. Starting from the recorded multiplexed image, shown in Figure 2a, this process benefits from the off-axis holographic configuration, where distinct holographic terms appear separated in the Fourier domain (Figure 2b). In the Fourier transform, holographic cross-correlation terms ($OR^{*}$ and $RO^{*}$) and the BF image (BFI) with the holographic auto-correlation term (${\left|O\right|}^{2}+{\left|R\right|}^{2}$) are located in different spectral regions, allowing the selective retrieval of the QPI- and BF-related frequency compounds.

To obtain the QPI image, the complex amplitude information of one of the holographic cross-correlation terms (e.g., $B=OR^{*}$, indicated by the green circle in Figure 2b) is extracted via previously reported Fourier filtering-based evaluation [43]. The recovered phase and intensity images are shown in Figure 2c and Figure 2d, respectively. In the intensity image of Figure 2d, it is hardly possible to distinguish the cells from the background due to the weak diffraction of in-focus cells and the presence of coherence-induced image disturbances. This issue underlines the benefit of complementary captured BF images under incoherent illumination. We can see that the recovered phase (Figure 2c) is distorted by an experimental setup-specific spherical phase aberration, which was corrected using a previously reported phase aberration compensation method [49]. Subsequently, phase unwrapping and background subtraction algorithms are applied to achieve the final QPI image (Figure 2e). This method is advantageous for imaging dense samples, such as cell cultures or tissue sections, since it requires no prior knowledge of the setup and/or specimen properties and numerical fitting [49].

The BF image recovery employs the SC-BF-SMIM procedure [43]. Fourier filtering was applied to the central Fourier transform region (blue circle in Figure 2b), which contains spatial frequencies from the holographic auto-correlation term and BF image ($A={\left|O\right|}^{2}+{\left|R\right|}^{2}+BFI$) (Figure 2f). To achieve the final BF image, the influence of ${\left|O\right|}^{2}+{\left|R\right|}^{2}$ is minimized by subtracting the weighted cross-correlation term as:

(2) $$BFI=|A|-w\left|B\right|,$$

where w is a weight factor ranging between 0 and 1 [43]. Since *w* ∈ [0, 1] and $\left|A\right|\geq \left|B\right|$, the subtraction operation does not produce negative values during the calculation of BFI. The subtraction in Equation (2) yields a noise-reduced BF image, as depicted in Figure 2g. The optimization of the parameter w for minimal noise in the BF image is detailed in Section 3.1.

### 2.3. Sample Preparation

For the experimental characterization of Section 3.1, we used poly(methyl methacrylate) (PMMA) microspheres (PolyAn GmbH, Berlin, Germany, 10 µm diameter, refractive index of 1.48) [50]. The microspheres were suspended in a 70/30 glycerin–water solution (refractive index of 1.43, measured with an Abbe refractometer) and mounted between a microscope slide and a coverslip.

Pancreatic tumor cells (PaTu 8988 T) and mouse fibroblasts (NIH-3T3) were cultivated according to standard cell culture procedures without antibiotics in Dulbecco’s Modified Eagle Medium (DMEM, Sigma Aldrich, St. Louis, MO, USA), supplemented with 1mM Pyruvate (Merck, Darmstadt, Germany), 2 mM Glutamine (Merck, Darmstadt, Germany), and 10% fetal calf serum (FCS, PAN Biotech, Aidenbach, Germany). NIH-3T3 mouse fibroblasts were passaged three times a week. In contrast, PaTu 8988 T cells were cultured with 5% horse serum (FCS, PAN Biotech, Aidenbach, Germany) and 5% fetal calf serum (FCS, PAN-Biotech, Aidenbach, Germany) and passaged twice a week. Mycoplasma contamination was routinely monitored using a commercial qPCR kit (Sartorius, Göttingen, Germany).

For BF-DHM measurements, cells were cultivated until reaching 90% confluence, then harvested using trypsin/EDTA (Thermo Fisher Scientific, Waltham, MA, USA), pelleted at 300× *g* for 5 min, and resuspended in filtered cell culture medium. NIH-3T3 cells were seeded at a density of 50,000 cells/mL in a volume of 3 mL into imaging dishes (35 mm µ-Dish ibiTreat, ibidi, Munich, Germany), while adherent PaTu 8988 T cells were seeded at a density of 5000 cells/mL and subconfluently observed in cell culture medium using object carrier slides and cover slips with the help of spacers (SecureSeal™ Imaging Spacer-SS1X9, 9 mm Diameter ID X 0.12 mm Depth, Grace Biolabs, Bend, OR, USA). Cell densities were automatically determined using a label-free digital holography-based device (Fluidlab R-300, Anvajo, Dresden, Germany). Cells were incubated for 24 h at 37 °C and 5% CO_2_. For experiments with suspended cells, PaTu 8988 T cells were suspended in DMEM at a concentration of 1.5 × 10^6^ cells/mL and analyzed in a microfluidic chip.

### 2.4. Illumination Conditions

To ensure precise control of the illumination conditions, the emission powers of both light sources of the BF-DHM microscope—the laser (for coherent illumination) and the LED (for incoherent BF illumination)— were measured at the sample plane using a power meter (LabMax™-TOP Laser Power/Energy Meter, Coherent, OR, USA) with a detection area of 50 mm^2^. The emission power settings used in the experiments were precisely controlled, with $P_{LASER}$ ranging from 0.2 mW to 2.0 mW in 0.2 mW increments, and $P_{LED}$ varying from 3 mW to 30 mW in 3 mW steps.

### 2.5. Microfluidic System

The microfluidic system utilized a syringe pump (Nemesys, CETONI GmbH, Korbußen, Germany) to guide cells through the microfluidic channel. This channel was fabricated using conventional soft lithography techniques in polydimethylsiloxane (PDMS, Dow Corning, Midland, MI, USA). The PMDS structure, which had a thickness of 1 mm, was bonded to a glass coverslip (175 µm thick) through air–plasma activation, following previously established protocols [51,52]. To enable lateral hydrodynamic focusing, the microfluidic chip was designed with a co-flow architecture. It featured a dedicated inlet for sample fluid at the center, while the sheath fluid was split into two streams at the lateral borders. These sheath fluid channels entered the system at a 30 deg angle relative to the sample fluid channel and had a rectangular cross-section of 1 mm width and 20 µm height.

## 3. Experimental Results

### 3.1. Multimodal Imaging Characterization

To validate the multimodal system for accurate QPI and noise-reduced BF imaging, a systematic characterization was performed using 10 µm PMMA microspheres, focusing on key parameters such as the weight factor (w), laser emission power ($P_{LASER}$), and LED emission power ($P_{LED}$). These parameters are critical for imaging quality and have to be carefully adjusted for maximized performance. For system characterization, microspheres were used. Microspheres are widely employed in microscopy due to their well-defined properties and their ability to serve as reliable reference materials. Due to the limited availability of well-characterized calibration specimens with a refractive index similar to living cells (approx. 1.38), PMMA microspheres were utilized in our study. PMMA microspheres are biocompatible and have a refractive index of 1.48, which is moderately close to the cellular refractive index. The size of 10 µm was chosen as a compromise that reflects the dimensions of the investigated cellular specimens and addresses the demands concerning the characterization of the experimental setup, including the FOV achieved with the utilized 20× microscope objective, as well as the corresponding resolution and calibration accuracy.

#### 3.1.1. Influence of the Weighting Factor in the Recovered BF Image

**Figure 3:**
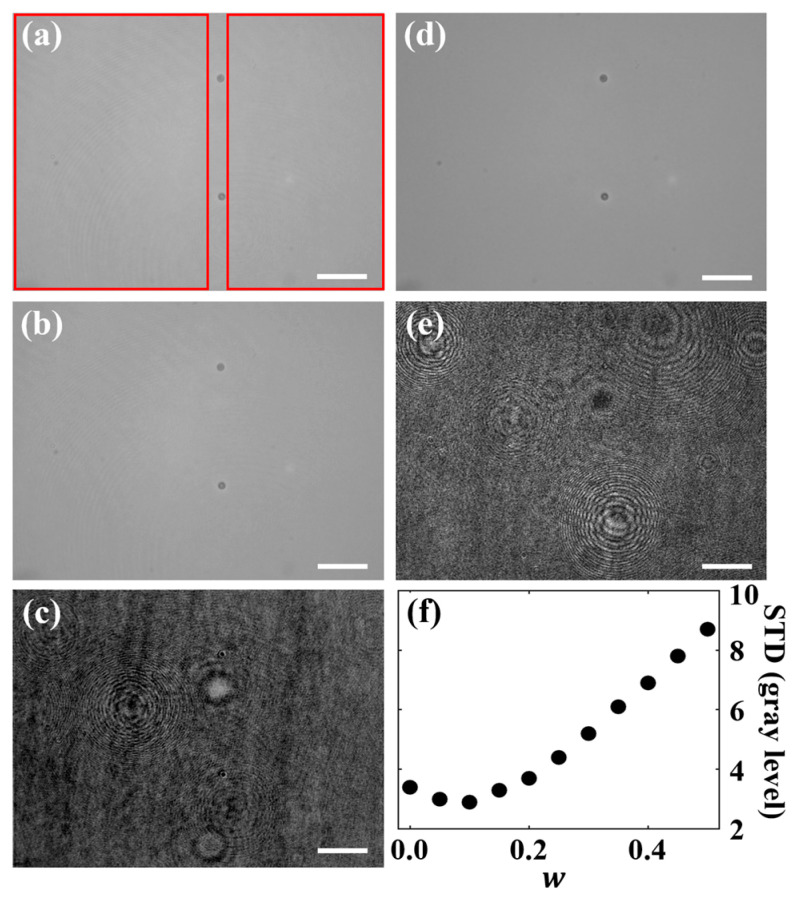
Influence of the weight factor w on the reconstructed BF images in the multimodal BF-DHM system for a laser light power of 0.4 mW and an LED light power of 30 mW). (**a**–**c**) BF images recovered with weighting values *w* = 0.0, 0.1, and 0.5, respectively. (**d**) Intensity image provided by separate BFM for visual comparison. (**e**) Intensity image recovered from a DHM off-axis hologram after Fourier filtering for visual comparison. (**f**) Influence of the weight factor w on the standard deviation (STD) values computed in the background regions without sample (marked with red rectangles in (**a**)), indicating noise levels in the reconstructed BF images. Scale bars correspond to 50 µm.

This section describes how the weight factor w influences the quality of the reconstructed BF images in the multimodal BF-DHM system. Figure 3 shows the influence of different w values in the BF image reconstruction for a camera exposure time of 0.9 ms, a laser emission power of 0.4 mW, and an LED emission power of 30 mW. Figure 3a–c depicts experimentally retrieved BF images that were reconstructed using w values ranging from 0 to 0.5, with a value interval of 0.05. These results are compared with separately recorded BF and DHM intensity images, shown in Figure 3d and Figure 3e, respectively, achieved by sequential illumination, thus also allowing a visual comparison with the multimodal approach.

The results in Figure 3a–c reveal that smaller w values (0.0 to 0.2) yield BF images that more closely resemble the ground truth BF image (Figure 3d). In contrast, larger w values (0.2 to 0.5) generate BF images (see Figure 3c) that present a stronger resemblance to the noisy intensity profile typical of DHM images that are recorded with coherent laser light (Figure 3e). These findings suggest that lower w values as more suitable for preserving the BF image quality, while higher values introduce coherence artifacts and noise, thereby reducing the overall image quality.

To quantitatively determine the optimum w for best BF image recovery, the standard deviation (STD) was calculated in sample-free background regions (within the red rectangles in Figure 3a) for w values ranging from 0.0 to 0.5. The results in Figure 3f show that higher weighting values are associated with an increased background STD, indicating noisier images, which is consistent with the visual observations from Figure 3a–e. Notably, the STD values follow a concave parabolic trend across the investigated range of w. The lowest STD (2.9 gray levels) occurs at w = 0.1, identifying it as the optimal weight factor ($w_{opt}$ = 0.1). On the other hand, the STD computed for the same background regions in the ground truth BF image (see Figure 3d) is 2.6 gray levels, which is slightly lower than the STD value achieved for the optimum w value. In contrast, the STD value computed for the DHM-only intensity image (see Figure 3e) is 25.6 gray levels, which exhibits an 8.8 times higher noise level than the BF images recovered by our BF-DHM system. This confirms the significant noise reduction of the BF images achieved by BF-DHM in comparison to DHM. The optimum w value aligns with the visual assessment, where the BF image at w = 0.1 (see Figure 3b) best matches the ground truth BF image (Figure 3d), confirming $w_{opt}$ = 0.1 as the ideal value for the given illumination conditions (see Figure 3a–d). However, other illumination conditions can induce other optimal *w* values (see Section 3.1.2).

#### 3.1.2. Influence of Laser Light Emission Power

**Figure 4:**
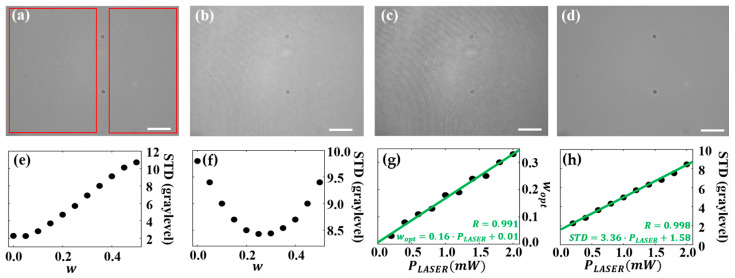
Influence of the laser light power ($P_{LASER}$) on the quality of recovered BF images for a bright-field illumination light power $P_{LED}$ = 30 mW. (**a**–**c**) BF images recovered at $P_{LASER}$ = 0.2 mW, 1.0 mW, and 2.0 mW, respectively. (**d**) Separately recorded BF image. (**e**,**f**) STD (computed in red-framed background regions without samples in (**a**)) as a function of the weighting factor w for $P_{LASER}$ = 0.2 mW and 2.0 mW, respectively. (**g**,**h**) $w_{opt}$ and STD vs. $P_{LASER}$, respectively, including the linear fits and the corresponding fitting equations and R values. Scale bars correspond to 50 µm.

In this section, we analyze the impact of laser emission power ($P_{LASER}$) on the quality of the QPI and BF image reconstructions. Multiplexed images were captured at various $P_{LASER}$ settings, ranging from 0.2 mW to 2.0 mW, while keeping LED power constant at 30 mW. Figure 4 presents the reconstructed BF images. Figure 4a–c includes the BF images recovered at $P_{LASER}$ = 0.2 mW, 1.0 mW, and 2.0 mW, respectively, after considering optimal w values (0.05, 0.15, and 0.25). The overall noise increases with $P_{LASER}$, while the lowest $P_{LASER}$ (0.2 mW) provides a BF image closely matching with the ground truth BF image from a standard BFM (Figure 4d). These results confirm that $P_{LASER}$ directly affects BF image quality.

Laser light power $\;P_{LASER}$ also influences $w_{opt}$. To analyze this relation, the STD in background regions without samples (red rectangles in Figure 4a) were determined for each reconstructed BF image. In Figure 4e,f, the STD is plotted vs. w for $P_{LASER}$ = 0.2 mW and 2.0 mW, respectively. The study shows that increasing the value of $P_{LASER}$ from 0.2 mW to 2.0 mW shifted the optimal $w_{opt}$ from 0.05 to 0.25, and accordingly, also the corresponding STDs of the image background from 2.25 to 8.45 gray levels. The observed shifts indicate a stronger presence of the holographic intensity component at higher values of $P_{LASER}$, which aligns with the visual observations. To quantify this effect, we plot $w_{opt}$ as a function of $P_{LASER}$ (Figure 4g), as well as the corresponding STDs (considering $w_{opt}(P_{LASER})$) (Figure 4h). The linear fits in those plots reveal a linear increase of both parameters with $P_{LASER}$, whose fitting equations are $w_{opt}=0.16\cdot P_{LASER}+0.01$ and $STD=3.36\cdot P_{LASER}+1.58$, respectively. This shows that the BF image reconstruction method does not eliminate the holographic intensity image completely but minimizes the contribution of coherence-induced image disturbances, as described in [43]. The highest BF image quality is achieved at the lowest $P_{LASER}$. In our study, the best match with the ground truth BF image (STD = 1.84 gray levels) was achieved for $P_{LASER}$ = 0.2 mW, with an STD of 2.25 gray levels. Hence, $P_{LASER}$ = 0.2 mW was identified to be optimal for BF image recovery in this setup, providing the highest noise reduction in the recovered BF images.

**Figure 5:**
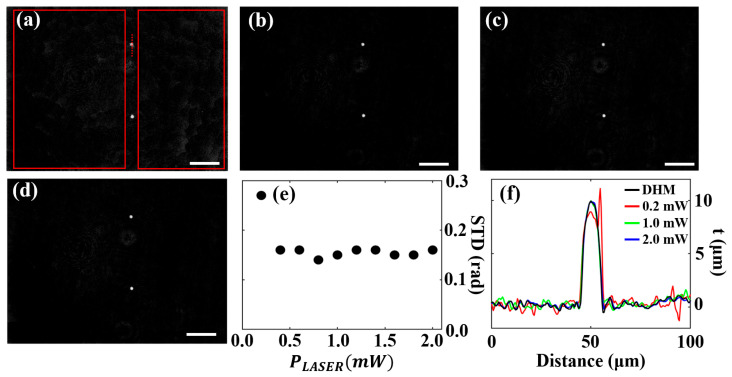
Influence of $P_{LASER}$ on the quality of the recovered QPI images for an LED emission power of 30 mW. (**a**–**c**) Recovered QPI images for $P_{LASER}$ = 0.2 mW, 1.0 mW, and 2.0 mW, respectively. (**d**) Separately recorded DHM QPI image serving as ground truth image. (**e**) STD computed in background regions (red-framed rectangles in (**a**)) of the recovered QPI images without a sample vs. $P_{LASER}$. (**f**) Color-coded thickness profiles along the dotted line in (**a**), calculated using Equation (3). Scale bars correspond to 50 µm.

In Figure 5, the influence of $P_{LASER}$ on the quality of the recovered QPI images is analyzed. Figure 5a–c includes three exemplary recovered QPI images that were acquired with different laser powers: $P_{LASER}$ = 0.2 mW, 1.0 mW, and 2.0 mW, respectively. For $P_{LASER}$ = 0.2 mW, the QPI image shows an obviously enhanced background noise. In contrast, the images recovered for $P_{LASER}$ = 1.0 mW and 2.0 mW show smoother backgrounds, which align with the separately captured reference DHM QPI image (Figure 5d). In addition, the quality of the recovered BF-DHM QPI images was assessed by quantifying background noise and the accuracy of phase values. Background noise was characterized by measuring STDs, as explained for Figure 3. The influence of $P_{LASER}$ on the QPI image STD is plotted in Figure 5e. Except for the lowest emission power (0.2 mW), which showed a significantly increased STD, all other values of $P_{LASER}$ yielded similarly low STD. This indicates a stable QPI performance regarding background noise for $P_{LASER}$ values above 0.2 mW. In addition, the STD values for the higher $P_{LASER}$ levels range from 0.14 to 0.16 rad, which are only slightly higher than the STD for the separately recorded DHM QPI image (0.13 rad).

The accuracy of the determined phase values was assessed by examining phase profiles along a microsphere (indicated by the dotted line in Figure 5a). Figure 5f shows the corresponding color-coded thickness *t* profiles derived from the phase profiles [53]:

(3) t=φ·λ/2πΔn,

where *φ* represents the phase, λ is the laser wavelength (532 nm), and Δn is the RI difference between the microsphere material and the surrounding water–glycerol immersion medium. The thickness profiles for the different $P_{LASER}$ values closely match the profile from the separately recorded DHM QPI image, except for $P_{LASER}$ = 0.2 mW. This indicates that the recovered QPI images provide accurate phase information comparable to separately captured QPI images, provided the illumination power exceeds a certain threshold.

In summary, the multimodal imaging system demonstrated distinct performance characteristics for QPI and BF imaging at varying laser power levels. Noise-reduced BF images were produced at low $P_{LASER}$, with image quality progressively deteriorating at higher $P_{LASER}$ due to the higher presence of coherence-induced image disturbances. For QPI images, the background noise remained almost constant across most $P_{LASER}$ values, except for the lowest laser light power (0.2 mW), which exhibited increased noise. A laser light power of 0.2 mW provided a multiplexed image with low hologram contrast. Poor off-axis fringe contrast leads to a weak QPI signal, making it difficult to distinguish sample-induced phase variations from background noise. This induces phase-unwrapping errors and background artifacts in the recovered QPI image, as shown in Figure 5a, degrading QPI image quality. In addition, QPI images retained accurate phase values across various $P_{LASER}$ settings, providing results comparable to conventional DHM. However, to achieve sufficient hologram contrast for effective QPI recovery, a minimum $P_{LASER}\;$= 0.4 mW was determined to achieve optimum QPI image quality in the applied experimental setup. Hence, the optimal $P_{LASER}$ setting balances BF and QPI image recovery: $P_{LASER}$ = 0.2 mW minimizes BF image noise, while $P_{LASER}=$0.4 mW ensures accurate QPI reconstruction. We selected $P_{LASER}$ = 0.4 mW as the best tradeoff for both.

#### 3.1.3. Influence of LED Light Emission Power

**Figure 6:**
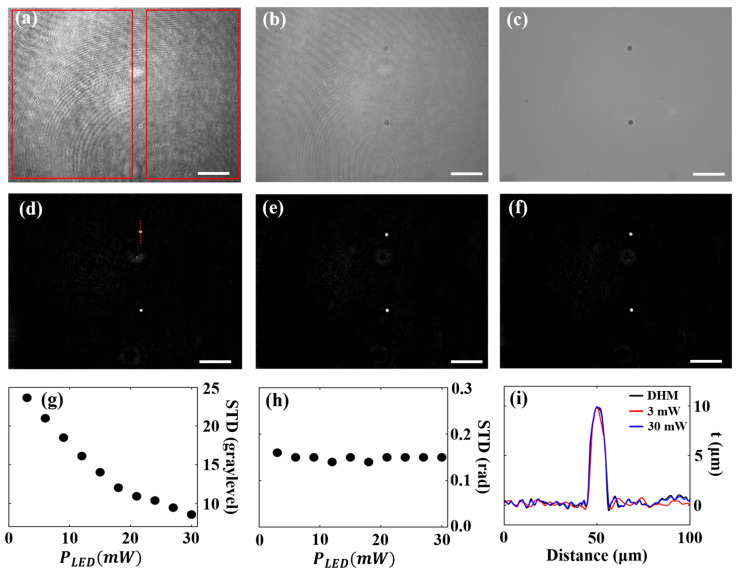
Influence of $P_{LED}$ on the quality of the recovered BF and QPI images for $P_{LASER}$ of 2.0 mW. (**a**,**b**) Recovered BF images for $P_{LED}$ = 3 mW and 30 mW, respectively. (**c**) Separately captured BF image serving as ground truth. (**d**,**e**) Recovered QPI images for $P_{LED}$ = 3 mW and 30 mW, respectively. (**f**) Separately captured DHM QPI image serving as ground truth. (**g**,**h**) STD values computed in the background regions (red-framed rectangles in (**a**)) without samples of the recovered BF (in gray levels) and QPI (in radians) images as a function of $P_{LED}$, respectively. (**i**) Thickness profiles along the dotted lines included in (**d**–**f**), calculated using Equation (3). Scale bars correspond to 50 µm.

In this section, the impact of $P_{LED}$ on the imaging performance of the multimodal imaging system is analyzed. Therefore, multiplexed images were recorded at a constant laser light power of $P_{LASER}$ = 2.0 mW, while the LED light power $P_{LED}$ was stepwise increased from 3.0 mW to 30.0 mW. Figure 6 presents the resulting recovered BF and QPI images. Figure 6a and Figure 6b show representative BF images recorded for $P_{LED}$ = 3 mW and 30 mW, respectively. Figure 6c shows the separately recorded BF image, serving as a ground truth. The comparison of the images illustrates the influence of $P_{LED}$ on the quality of the reconstructed BF images. In this analysis, a higher $P_{LASER}$ was used compared to previous sections, which increases the visibility of coherence-induced noise in the recovered BF images in comparison to Figure 3 and Figure 4. In addition, Figure 6d,e displays the recovered QPI images for $P_{LED}$ = 3 mW and 30 mW, respectively. The recovered QPI images exhibit a similar quality as the separately recorded DHM QPI image (Figure 6f). Subsequently, the STD of the background within the sample-free regions (marked in Figure 6a) and the accuracy of the phase values were assessed. Figure 6g,h presents the STD values for BF (in gray levels) and QPI imaging (in radians) as a function of $P_{LED}$, respectively. Figure 6g indicates that an increase of $P_{LED}$ leads to a reduction in the background STD of the BF images. This suggests that higher $P_{LED}$ improves BF image quality by enhancing the BF contribution relative to the holographic intensity image. In Figure 6h, the STD values of the QPI image background remain approximately constant across increasing $P_{LED}$ values. The stability of the STD implies that QPI image quality is not affected by variations in $P_{LED}$, resulting in consistent performance regardless of the intensity of the bright-field illumination. In addition to the background noise analysis, Figure 6i presents the thickness profiles derived from the phase profiles along (designated line in Figure 6d) using Equation (3). The profiles confirm the accuracy of QPI, with thickness values for the PMMA microspheres closely aligning with those from the separately recorded DHM QPI image for all $P_{LED}$ values. According to these findings, the optimal value is identified as $P_{LED}$ = 30 mW, as this setting minimizes the background STD in the BF images while maintaining QPI quality comparable to separately captured DHM QPI images, and therefore, enhancing BF quality without compromising QPI accuracy.

### 3.2. Multimodal Bright-Field and DHM-Based QPI of Adherent Cells

**Figure 7:**
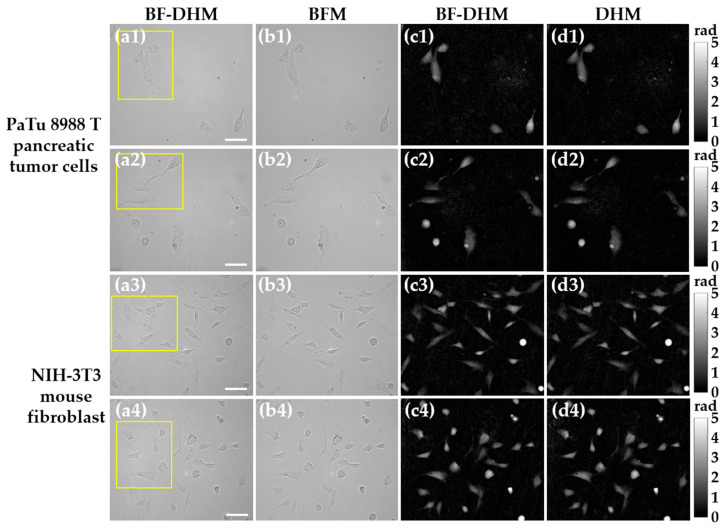
Multimodal imaging of living adherent cells using optimum light power parameters ($P_{LED}$ = 30 mW, $P_{LASER}$ = 0.4 mW). Rows (**1**) and (**2**) show experimental results for different ROIs of adherent pancreatic tumor cells (PaTu 8988 T), while rows (**3**) and (**4**) show experimental results for different ROIs of adherent mouse fibroblasts (NIH-3T3). Columns (**a**,**b**) show BF images recovered from the BF-DHM and a separately recorded BFM image, respectively. Columns (**c**,**d**) show QPI images recovered from the BF-DHM and separately recorded DHM QPI images, respectively. Yellow rectangles indicate ROIs, which are depicted enlarged in Figure S1 of Supplementary Materials to highlight small cellular details. Phase scale bars on the right apply to both QPIs in columns (**c**,**d**). Scale bars correspond to 50 µm.

We further evaluated the proposed BF-DHM system for imaging of living, adherently grown pancreatic tumor cells (PaTu 8988 T) and mouse fibroblasts (NIH-3T3), due to their structural, biological, and optical properties [21,54,55]. Pancreatic tumor cells display compact and irregular morphologies, while fibroblasts exhibit a more elongated morphology. In addition, fibroblasts play a crucial role in maintaining tissue structure and function, while pancreatic tumor cells were selected due to their clinical relevance, as pancreatic cancer is highly aggressive and challenging to detect in its early stages, making it a critical target for improved imaging techniques. Cells were imaged at room temperature in cell culture medium using microscope slides and coverslips, with the optimal illumination settings: $P_{LASER}$ = 0.4 mW and $P_{LED}$ = 30 mW. Figure 7 presents exemplary results from different regions of interest (ROIs), shown in four different rows [rows (1) and (2) correspond to pancreatic tumor cells, and rows (3) and (4) show the results for mouse fibroblasts]. Enlarged images of the ROIs outlined in yellow rectangles in Figure 7a are shown in Figure S1 (Supplementary Materials). Figure 7a,b displays BF images obtained from the BF-DHM system and separately recorded BF images, respectively. The BF images from the BF-DHM system were observed to be slightly noisier than those from the separately recorded BF images. This additional noise can be explained by the residual effects of coherent illumination, which were not entirely removed by the image reconstruction process. Despite the noise, the BF-DHM system was able to capture the same fine details as visible in the separately recorded BFM image. The equivalence of the image content is highlighted in the magnified ROIs shown in Figure S1a,b, where both methods resolve essential cellular structures (such as nuclei, nucleoli, and filopodia) without losing fine details (see green arrows in Figure S1a,b). In addition, Figure 7c,d provides the QPI images recovered by the BF-DHM system and separately recorded DHM QPI images, respectively. The QPI images produced by the BF-DHM system show quality comparable to separately recorded DHM QPI, as verified by examining the magnified images in Figure S1c,d. This similarity suggests that the BF-DHM is an effective system for QPI imaging without compromising quality relative to established DHM methods. By comparing the BF images (Figure S1a,b) with the QPI images (Figure S1c,d), the study highlights how BF imaging can reveal fine cellular components and organelles that are not discernible in QPI, as indicated by green (discernible) and red (non-discernible) arrows. Additionally, our concept also permits the generation of a merged pseudo-3D representation of the QPI image (Figure S1c) and the BF image texture (Figure S1a), as shown in Figure S1e. This underscores the significance of having both imaging modalities simultaneously from a single camera snapshot, allowing collocated examination of both structural and quantitative phase details of cells.

### 3.3. Multimodal Bright-Field and DHM-Based QPI of Cells in Flow

**Figure 8:**
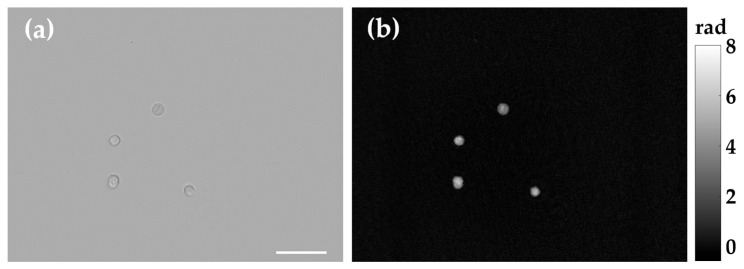
Representative image from a movie sequence (Video S1) retrieved by the proposed BF-DHM system. Living suspended cells (PaTu 8988 T) were imaged while flowing through a microfluidic chip, using optimum light power parameters ($P_{LED}$ = 30 mW, $P_{LASER}$ = 0.4 mW). (**a**) Recovered BF image. (**b**) Recovered QPI image. Scale bar represents 50 µm.

Finally, the single-shot capability of our BF-DHM concept was evaluated for multimodal imaging of fast-moving objects. Living suspended PaTu 8988 T cells were observed in flow within a microfluidic chip (see Section 2.5). For the experiments, PaTu 8988 T cells were suspended in DMEM at a concentration of 1.5 × 10^6^ cells/mL. The cells were examined with our BF-DHM system under laminar flow conditions, with flow rates set at 10 μL/min for the sheath fluid and 2 μL/min for the sample fluid. Multiplexed images were captured over a period of 5 s, with an acquisition rate of 47 fps and an exposure time of 0.9 ms. Exemplary results are presented in Figure 8 and Video S1. Figure 8a,b shows representative BF and QPI images recovered from a recorded image stack. Figure 8 and Video S1 demonstrate reliable, simultaneous single-shot BF imaging and QPI of fast-moving objects without motion artifacts, which validate our concept for label-free multimodal imaging flow cytometry.

## 4. Discussion and Conclusions

In this contribution, we presented a single-capture multimodal BF-DHM system based on a Mach–Zehnder interferometer. Our multimodal imaging approach was implemented based on a commercial inverted BF light microscope with an added external fiber-based off-axis DHM module. The setup allowed us to obtain accurate QPI and noise-reduced BF images from a single multiplexed capture acquired with a monochrome digital camera sensor. The numerical demultiplexing approach involved Fourier filtering, aberration compensation, and weighted image subtraction procedures [43,49]. Additionally, we conducted a systematic characterization of the system’s multimodal imaging performance by evaluating the impact of variations in critical optical parameters such as coherent and incoherent illumination light powers. The optimization process depends on the experimental setup but only needs to be performed once during the initial calibration for a specific configuration. This optimization process does not depend on the dynamic range of the camera or sample properties such as thickness, refractive index, or absorption. In our study, $P_{LASER}$ and $P_{LASER}$ were determined for our individual experimental setup with specific laser and LED sources, aiming to achieve low exposure times (0.9 ms) for fast multimodal imaging. However, other multimodal systems may require different values for $P_{LASER}$ and $P_{LED}$ depending on the specific hardware configuration and application in biological imaging. To validate the performance, we first optimized the system using a sample containing microspheres and then evaluated it for multimodal imaging of various biological samples. Results from experiments with living adherent human pancreatic tumor cells and mouse fibroblasts allowed us to assess the performance of the multimodal microscopy system for capturing cellular specimens with different morphologies, while data from investigations conducted with suspended cells in flow within a microfluidic system validated the proposed concept for multimodal imaging of fast dynamic events. In our study, imaging was performed with a 20× microscope objective. However, BF-DHM can be also applied using objectives with other magnifications depending on the purpose of the intended application. Although validated for biological samples, the proposed system can be also applied to other types of samples.

Regarding the characterization of the system, according to the experimental results achieved in Section 3.1, the multimodal imaging performance was optimized by using the lowest coherent light emission power required for accurate QPI (in our case, $P_{LASER}$ = 0.4 mW) and the highest incoherent light emission power (in our case, $P_{LED}$ = 30 mW). The main reasons were that, in the QPI reconstruction process, the incoherent illumination was not (or very marginally) involved, so only the coherent illumination emission power determined the QPI image quality and the accuracy for the detection of optical path length changes. In addition, as shown in Figure 5, the quality and accuracy of the QPI reconstructions remained approximately constant for all laser light power values higher than or equal to 0.4 mW, independently from the value of the incoherent light emission power. Hence, independently of the incoherent illumination emission power, accurate and high-quality QPI images were reconstructed when the coherent illumination emission power was high enough to register an appropriate interference pattern. However, the holographic intensity images, and thus, the coherent illumination emission powers, were directly involved in the reconstruction of the BF images, increasing the image noise with the coherent illumination emission power, and therefore decreasing the quality of the BF reconstructions when increasing the coherent emission power. As a result, noise-reduced BF images were achieved when the ratio between incoherent/coherent illumination emission powers was maximum. Hence, optimum BF and QPI reconstruction is achieved when the ratio between incoherent and coherent illumination light emission powers is maximized, as long as the recorded multiplexed image contains a digital hologram with sufficient contrast in the off-axis carrier interference fringe pattern for QPI reconstruction.

Compared to SC-BS-SMIM systems, our multimodal approach introduces higher complexity, increasing alignment demands, and is more susceptible to reconstruction errors caused by vibrations and thermal changes, due to its double-path configuration [56]. Unlike a multimodal fluorescence-DHM system proposed by Nygate et al. [32], our approach does not entirely remove the holographic intensity image from the recovered BF image, leaving a slightly visible residual. However, this issue does not affect the spatial resolution of BF-DHM compared with separately recorded BF images (Supplementary Materials, Figure S2) and, as shown in Figure 7 and Figure S1, fine cellular details captured in sequential BF images (but are absent in QPI images) are still detectable in the recovered BF images. In addition, unlike sequential illumination and multi-camera BF-DHM setups, our single-capture method relies on dynamic range and spatial frequency multiplexing [57]. This restricts the digital camera’s dynamic range and the available spatial frequency in the Fourier domain for each imaging mode, which may pose challenges when imaging highly absorbing samples or if the digital sensor’s pixel size is not optimally matched to the system’s resolution and magnification. Compared to the system reported in [48], the proposed concept represents a further development since it captures both imaging modalities BF and DHM at the same time, providing the same information as systems based on sequential illumination and recording, but in a much faster way.

In conclusion, the proposed single-capture double-path BF-DHM system offers a simple and fast solution for label-free multimodal QPI-BF imaging, overcoming the limitations of previous BF-DHM systems that rely on sequential illumination, multiple cameras, or specific sample constraints. Our systematic analysis shows that, with optimized illumination settings, this system can provide accurate QPI and noise-reduced BF images from a single multiplexed capture. This study may serve as a reference for future development and application of other multimodal BF-DHM systems. Our experimental results highlight this single-capture double-path BF-DHM as a promising tool for biomedical applications requiring high acquisition rates, such as high throughput imaging of cells in well plates [58], imaging flow cytometry [59], or sperm motility analysis [60], as well as for analyzing large and connected samples in label-free histology [61].

## Data Availability

Data are available on request due to privacy restrictions.

## Supplementary Materials

The following supporting information can be downloaded at: https://www.mdpi.com/article/10.3390/s25092675/s1, Figure S1: Multimodal live-cell imaging using optimum light power parameters ($P_{LED}$ = 30 mW, $P_{LASER}$ = 0.4 mW). Images correspond to the ROIs enclosed in yellow rectangles in Figure 7. Rows (1) and (2) show the enlarged images of adherent pancreatic tumor cells (PaTu 8988 T), and rows (3) and (4) show the enlarged images of adherent mouse fibroblasts (NIH-3T3). (a) and (b) show BF images recovered from the BF-DHM and separately recorded BFM images, respectively. (c) and (d) show QPI images recovered from the BF-DHM and separately recorded DHM QPI images, respectively. (e) Perspective views integrating QPI values from BF-DHM (c) and BF image texture from (a). Color-coded arrows indicate small details that can (green) be resolved in the BF images but not (red) QPI images. Phase scale bars apply to both QPIs in columns (c) and (d). Scale bars represent 50 µm; Figure S2: Comparison of lateral resolution provided by (a) the proposed BF-DHM concept and (b) a separately recorded BF image utilizing a test chart with included cell phantoms and USAF test target-like line structures [62]. The comparison of the enlarged color-framed images shows that the lateral resolution is the same in both cases. Scale bars correspond to 50 µm; Video S1: Movie sequence retrieved by the proposed BF-DHM system. Living suspended cells (PaTu 8988 T) were imaged while flowing through a microfluidic chip, using optimum light power parameters ($P_{LED}$ = 30 mW, $P_{LASER}$ = 0.4 mW). (**a**) Recovered BF image. (**b**) Recovered QPI image.
