# Supplementary material for: Characterization of a Single-Capture Bright-Field and Off-Axis Digital Holographic Microscope for Biological Applications
Source: Sensors (Basel). 2025 Apr 23;25(9):2675. doi: 10.3390/s25092675 (PMC12074500; doi:10.3390/s25092675)
Supplement: Supplementary file 1 [file sensors-25-02675-s001.zip › sensors-3481305-supplementary.pdf]

Supplementary Materials

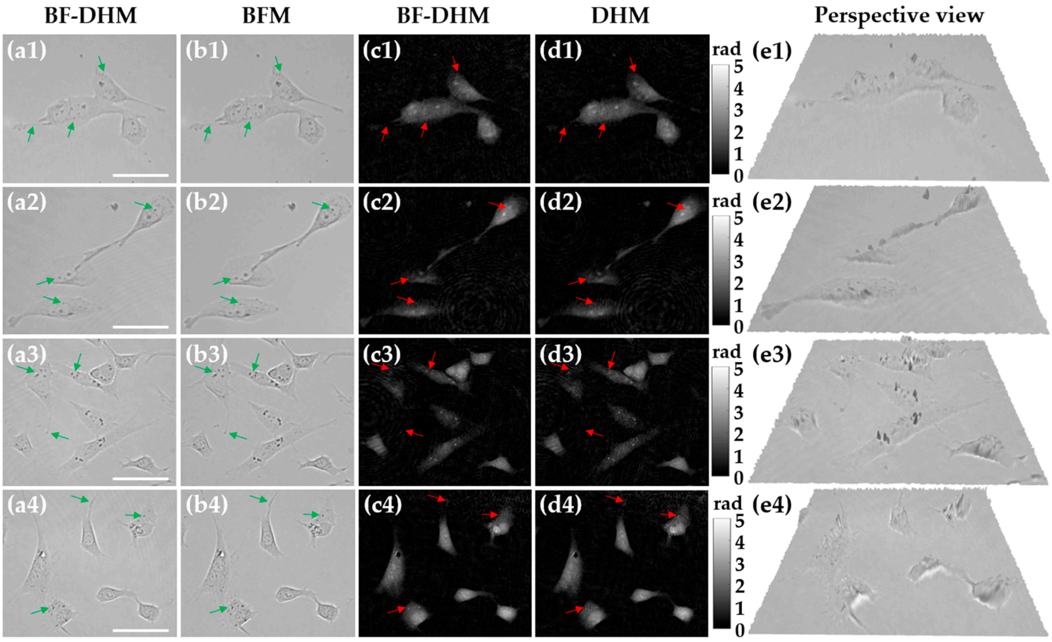

Figure S1. Multimodal live cell imaging using optimum light power parameters ($\boldsymbol{P_{LED}}$ = 30 mW, $\boldsymbol{P_{LASER}}$ = 0.4 mW). Images correspond to the ROIs enclosed in yellow rectangles in Figure 7. Rows (1) and (2) show the enlarged images of adherent pancreatic tumor cells (PaTu 8988 T), and rows (3) and (4) show the enlarged images of adherent mouse fibroblasts (NIH-3T3). (a) and (b) show BF images recovered from the BF-DHM and separately recorded BFM images, respectively. (c) and (d) show QPI images recovered from the BF-DHM and separately recorded DHM QPI images, respectively. (e) Perspective views integrating QPI values from BF-DHM (c) and BF image texture from (a). Color-coded arrows indicate small details that can (green) be resolved in the BF images but not (red) QPI images. Phase scale bars apply to both QPIs in columns (c) and (d). Scale bars represent 50 μm.

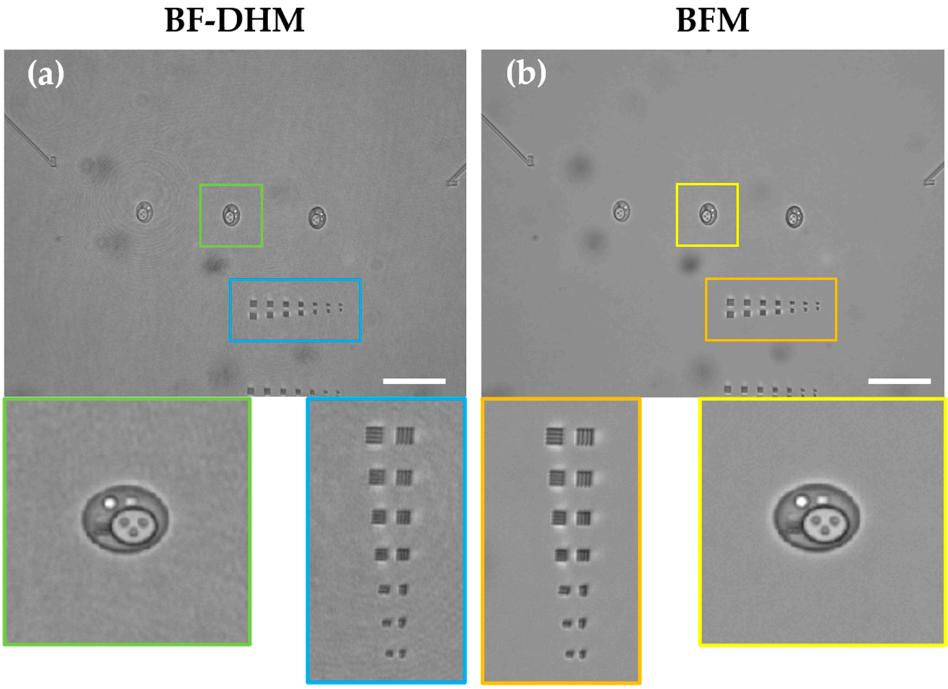

Figure S2. Comparison of lateral resolution provided by (a) the proposed BF-DHM concept and (b) a separately recorded BF image utilizing a test chart with included cell phantoms and USAF test target-like line structures [62]. The comparison of the enlarged color-framed images shows that the lateral resolution is the same in both cases. Scale bars correspond to 50 μm.
